# Genome Sequence of Bjerkandera adusta Strain Dec 1, a Basidiomycete Secreting DyP-Type Peroxidase

**DOI:** 10.1128/mra.01049-22

**Published:** 2023-01-04

**Authors:** Kanako Sugawara, Yasushi Sugano

**Affiliations:** a Department of Clinical Laboratory Sciences, Faculty of Health Sciences, Nihon Institute of Medical Science, Saitama, Japan; b Department of Chemical and Biological Sciences, Faculty of Science, Japan Women’s University, Tokyo, Japan; Vanderbilt University

## Abstract

We report the draft genome sequence of Bjerkandera adusta Dec 1, a basidiomycete that was isolated from the soil in Yokohama, Japan, using the Illumina HiSeq platform. B. adusta Dec 1 was identified as a fungus that degrades persistent anthraquinone dyes, and the novel peroxidase DyP was responsible for this degradation.

## ANNOUNCEMENT

Bjerkandera adusta, a basidiomycete belonging to the Polyporaceae family, is a wood-decaying white rot fungus that parasitizes certain woods. White rot fungi, including B. adusta, secrete lignin peroxidase (1), manganese peroxidase (2), and versatile peroxidase (3) to degrade the lignin in wood. B. adusta strain Dec 1, which was formerly classified as Geotrichum candidum (4), was isolated from a soil sample from Yokohama, Japan, and shown to efficiently degrade recalcitrant compounds, especially anthraquinone dyes, for which efficient bioremediation had not been reported previously (5, 6). Studies of the degradation of anthraquinone dyes by B. adusta Dec 1 led to the identification of DyP as a contributing enzyme (7).

We have studied the function of DyP and its physiological substrates in B. adusta Dec 1 (8). White rot fungi such as B. adusta possess a variety of secretory peroxidases and, in addition, our colleagues previously reported multiple isoforms of DyP from B. adusta Dec 1 (9); some of them seem to functionally complement each other. Thus, understanding the physiological function of DyP requires identification of all isoforms of DyP and, if necessary, performance of phenotypic analyses employing several strains that lack the gene encoding DyP. Although the genome sequence of the B. adusta HHB-12826 strain has been reported (10, 11), the genome of the B. adusta Dec 1 strain we used in our previous DyP studies was not sequenced, and little information is available on its isoforms. Therefore, in this study, we analyzed the B. adusta Dec 1 draft genome using the Illumina HiSeq platform.

Five milliliters of B. adusta Dec 1 suspension, including 1.0 × 10^7^ to 1.0 × 10^8^ spores/mL, was inoculated into 150 mL of fresh potato extract adjusted to pH 5.5. Cultures were grown at 29°C with shaking at 140 rpm (8). The collected mycelia were disrupted by freeze-drying and grinding. Genomic DNA was extracted from the disrupted mycelia using Quick-DNA fungal/bacterial kits (Zymo Research). The library for the Illumina platform was prepared using a TruSeq DNA PCR-free library preparation kit. The DNA was sheared into 350-bp fragments, and the fragments were subjected to end repair, A-tailing, and ligation. Genome sequencing was performed using a paired-end strategy and generated a total of 44,746,304 reads, with an average read length of 100.211 bp. For every read, adapter sequences and low-quality regions were trimmed using Cutadapt version 1.1 and Trimmomatic version 0.32, respectively, and *de novo* assembly was performed using Velvet version 1.2.10 (Illumina, Inc.). Gaps were closed using the gap-closing tool Platanus version 1.2.4. This resulted in a total scaffold length of 39,456,311 bp, distributed among 3,240 scaffolds. The longest scaffold was 1,323,668 bp, and the *N*_50_ value was 153,182 bp. The GC content of the genome was 54.3%.

### Data availability.

This genome sequencing project has been deposited in DDBJ/ENA/GenBank under the BioProject accession number PRJDB14086 (BioSample accession number SAMD00517220). The accession numbers of the sequence reads and the draft genome sequence are DRX383786 and BSCZ01000000, respectively. The strain Dec 1 was deposited in the National Institute of Technology and Evaluation with the registry number FERM P-15348.
